# Supplementary material for: Development of a genetically encoded sensor for probing endogenous nociceptin opioid peptide release
Source: Nat Commun. 2024 Jun 25;15:5353. doi: 10.1038/s41467-024-49712-0 (PMC11199706; doi:10.1038/s41467-024-49712-0)
Supplement: Supplementary file 1 — Supplementary Information [file 41467_2024_49712_MOESM1_ESM.pdf]

Supplementary Note 1

NOPLight1 DNA sequence:
ATGAAGACGATCATCGCCCTGAGCTACATCTTCTGCCTGGTGTTCGCCGACTACAAGGACGATGATG
ACGCCATGGAGCCACTTTTTCCGGCTCCCTTTTGGGAAGTTATCTATGGGTCTCACCTCCAGGGGAAT
TTGAGTCTTCTTAGCCCGAACCATTCTCTGCTGCCTCCACACCTTTTGCTGAACGCGTCACACGGTGC
CTTTCTTCCGCTCGGTCTTAAGGTAACCATTGTTGGACTCTATCTTGCTGTGTGCGTTGGAGGCTTGC
TGGGGAATTGTCTTGTCATGTATGTGATCTTGCGACATACCAAGATGAAGACTGCTACAAATATATA
CATATTTAATCTGGCACTTGCTGATACTCTTGTGTTGCTCACGCTGCCCTTTCAAGGGACTGACATAC
TCCTTGGGTTCTGGCCGTTCGGTAATGCACTCTGCAAAACCGTGATCGCAATTGATTATTACAATATG
TTTACGTCAACGTTTACTCTTACCGCTATGAGTGTGGATCGGTACGTCGCTATTTGTCACCCTAAACG
AGCGCTGGATGTTCGAACCAGTTCAAAAGCACAGGCCGTCAATGTAGCTATCTGGGCACTTGCAAG
TGTGGTCGGTGTGCCTGTGGCGATTATGGGGTCTGCGCAAGTAGAAGACGAAGAGATTGAGTGCTT
GGTTGAGATCCCTACACCCCAGGACTATTGGGGACCCGTATTCGCAATTTGTATTTTTCTCTTTTCTTT
TATTGTCCCCGTTCTGGTAATTTCTGTCTGTTATAGTCTCATGATACGCCGCCTGAGGGGGGTGCGCA
TAGCCCAGAAGAATAGCTCTCTCATCAACGTATATATAAAAGCCGATAAACAAAAGAATGGCATTAA
GGCGAATTTCAAGATTAGGCACAATATAGAAGACGGAGGTGTTCAACTTGCGTACCATTATCAGCA
GAATACCCCGATTGGGGATGGTCCTGTGCTTCTTCCCGACAATCACTATCTGAGCGTACAGAGCAAA
CTCTCCAAGGACCCGAACGAGAAACGGGATCACATGGTGCTGTTGGAATTTGTAACGGCAGCGGGG
ATTACTCTGGGCATGGACGAATTGTATAAAGGAGGCACGGGGGGTTCAATGGTTAGTAAAGGAGA
GGAACTTTTTACAGGCGTCGTCCCGATTTTGGTAGAATTGGATGGCGATGTAAACGGGCATAAATTT
AGTGTATCCGGCGAGGGAGAGGGAGATGCTACCTACGGGAAGCTCACCCTGAAATTCATCTGCACG
ACAGGTAAATTGCCTGTTCCGTGGCCGACTTTGGTGACGACTCTGACCTATGGTGTCCAGTGTTTCTC
TCGGTATCCAGACCATATGAAGCAGCATGACTTCTTCAAGAGCGCGATGCCAGAGGGTTACATTCAA
GAAAGGACTATATTCTTTAAAGATGACGGCAATTACAAGACTCGCGCTGAAGTTAAATTCGAAGGT
GATACATTGGTAAACCGAATAGAACTTAAGGGTATAGACTTCAAAGAAGATGGAAATATACTGGGC
CACAAACTTGAGTACAACAACCACGATCAGAAGGACAGAAATTTGCGACGGATCACGCGGCTCGTG
CTCGTTGTGGTTGCTGTTTTTGTTGGTTGTTGGACCCCGGTTCAGGTCTTTGTGTTGGCCCAAGGTCT
CGGGGTACAACCCAGCTCAGAAACAGCTGTCGCGATATTGAGGTTTTGTACGGCGCTTGGATACGT
CAACTCATGCTTGAACCCTATACTGTACGCTTTCCTCGACGAAAACTTTAAAAGGTGTTTTCGAGACT
TCTGCTTCCCGTTGAAGATGCGAATGGAGAGACAGGCCACTGCGAGAGTGCGGAATACGGTACAG
GACCCGGCGGCCCTCCGAGACATCGACGGAATGAACAAGCCTGTATAA

NOPLight1 protein sequence:
MKTIIALSYIFCLVFADYKDDDDAMEPLFPAPFWEVIYGSHLQGNLSLLSPNHSLLPPHLLLNASHGAFLPL
GLKVTIVGLYLAVCVGGLLGNCLVMYVILRHTKMKTATNIYIFNLALADTLVLLTLPFQGTDILLGFWPFGN
ALCKTVIAIDYYNMFTSTFTLTAMSVDRYVAICHPKRALDVRTSSKAQAVNVAIWALASVVGVPVAIMGS
AQVEDEEIECLVEIPTPQDYWGPVFAICIFLFSFIVPVLVISVCYSLMIRRLRGVRIAQKNSSLINVYIKADKQ
KNGIKANFKIRHNIEDGGVQLAYHYQQNTPIGDGPVLLPDNHYLSVQSKLSKDPNEKRDHMVLLEFVTA
AGITLGMDELYKGGTGGSMVSKGEELFTGVVPILVELDGDVNGHKFSVSGEGEGDATYGKLTLKFICTTG
KLPVPWPTLVTTLTYGVQCFSRYPDHMKQHDFFKSAMPEGYIQERTIFFKDDGNYKTRAEVKFEGDTLV
NRIELKGIDFKEDGNILGHKLEYNNHDQKDRNLRRITRLVLVVVAVFVGCWTPVQVFVLAQGLGVQPSSE
TAVAILRFCTALGYVNSCLNPILYAFLDENFKRCFRDFCFPLKMRMERQATARVRNTVQDPAALRDIDG
MNKPV*

Note: residues highlighted in red indicate positions mutated to alanine in NOPLight-ctr

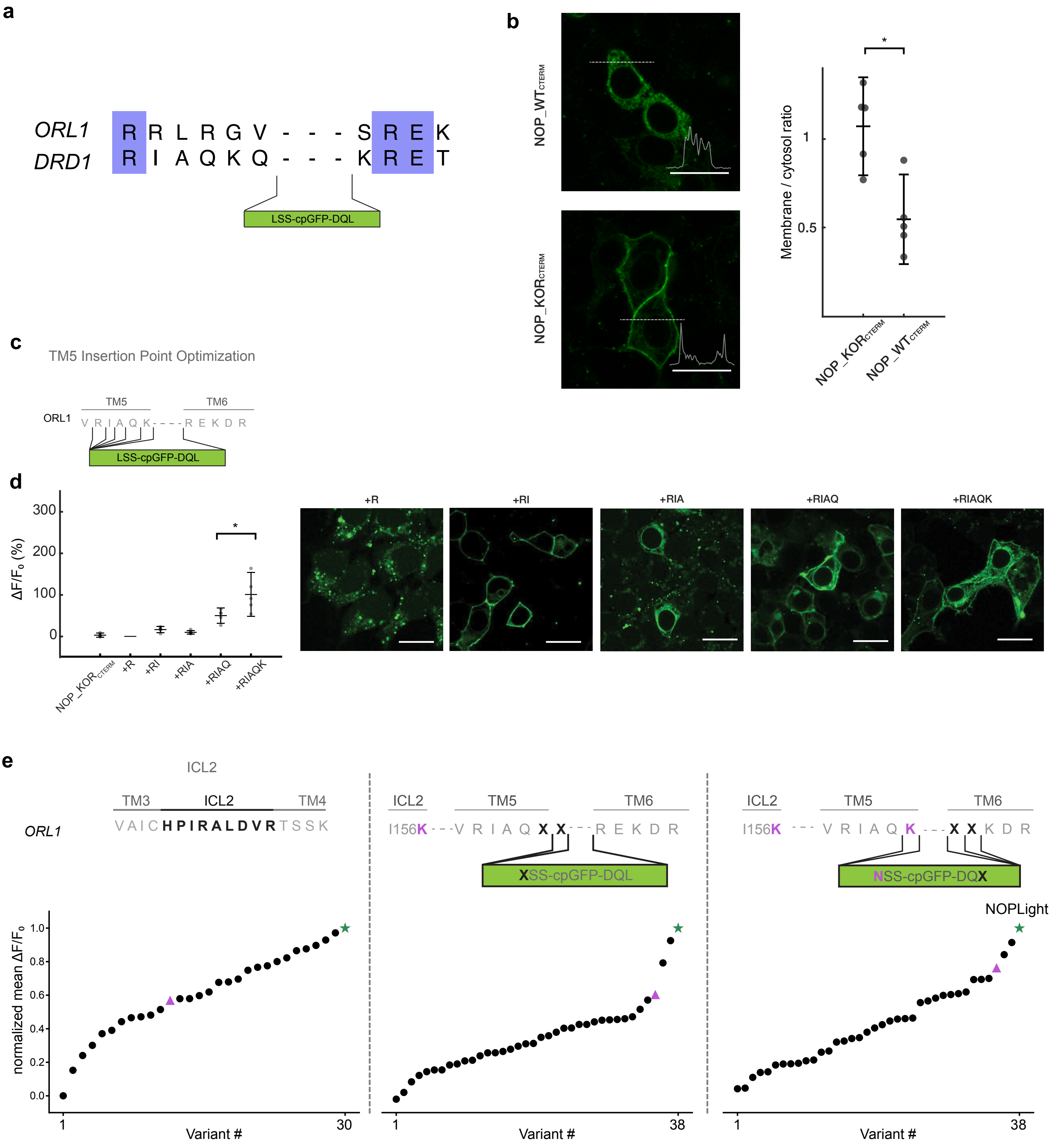

a
ORL1 R R L R G V - - - S R E K
DRD1 R I A Q K Q - - - K R E T
LSS-cpGFP-DQL
b
NOP_WTCTERM
NOP_KORCTERM
Membrane / cytosol ratio
1
0.5
*
NOP_KORCTERM
NOP_WTCTERM
c
TM5 Insertion Point Optimization
TM5
TM6
ORL1 V R I A Q K - - - - R E K D R
LSS-cpGFP-DQL
d
ΔF/F0 (%)
300
200
100
0
*
NOP_KORCTERM
+R
+RI
+RIA
+RIAQ
+RIAQK
e
ICL2
TM3 ICL2 TM4
ORL1
VAIC HPIRALDVR TSSK
ICL2 TM5 TM6
I156K - - - V R I A Q X X - - - R E K D R
XSS-cpGFP-DQL
ICL2 TM5 TM6
I156K - - - V R I A Q K - - - X X K D R
NSS-cpGFP-DQX
NOPLight
normalized mean ΔF/F0
1.0
0.8
0.6
0.4
0.2
0.0
1
30
38
Variant #

Supplementary Figure 1. Development and optimization of NOPLight.

a. Sequence alignment of transmembrane domains (TM) 5 and 6 from OPRL1 and DRD1 to determine the insertion site for the cpGFP module from dLight1.3. Color code indicates percent identity between the human NOPR and DRD1. **b.** Left: Representative images of membrane expression profile of HEK293T cells expressing *in silico* designed sensor prototype with a wild-type NOPR C-terminus ($N/OFQ_{\_WTcterm}$) and a chimeric sensor with the C-terminus of kappa opioid receptor ($N/OFQ_{\_KORcterm}$). Insets: fluorescent intensity of all pixels along the dotted line of each image. Scale bars, 10 µm. Right: Quantification of the ratio between pixel averaged fluorescent intensity of cell membrane and that of the cytosol. n = 5 cells. (P = 0.011, one-sided Mann Whitney U test) **c.** Schematic representation of the optimization of the insertion site at TM5 linker region. **d.** Left: Maximal $\Delta F/F_0$ response to 10 µM N/OFQ of all sensor prototypes shown in **c.** Right: representative image of HEK293T cells expressing sensor prototypes. **e.** Top: Schematic representation of three rounds of directed mutagenesis to improve sensor dynamic range. Amino acids that were mutated into a subset of different amino acids in the screening process are labelled in bold. Selected mutation from previous round of screening labelled in magenta. Bottom: Normalized $\Delta F/F_0$ response of HEK293T cells expressing each variant to 10 µM N/OFQ. Each data point represents averaged $\Delta F/F_0$ response from > 3 cells in 3 independent experiments normalized to the maximal $\Delta F/F_0$ response of the best variant in the current round of screening. Magenta: Selected variant from previous round of screening. Green: Best variant in current round of screening.

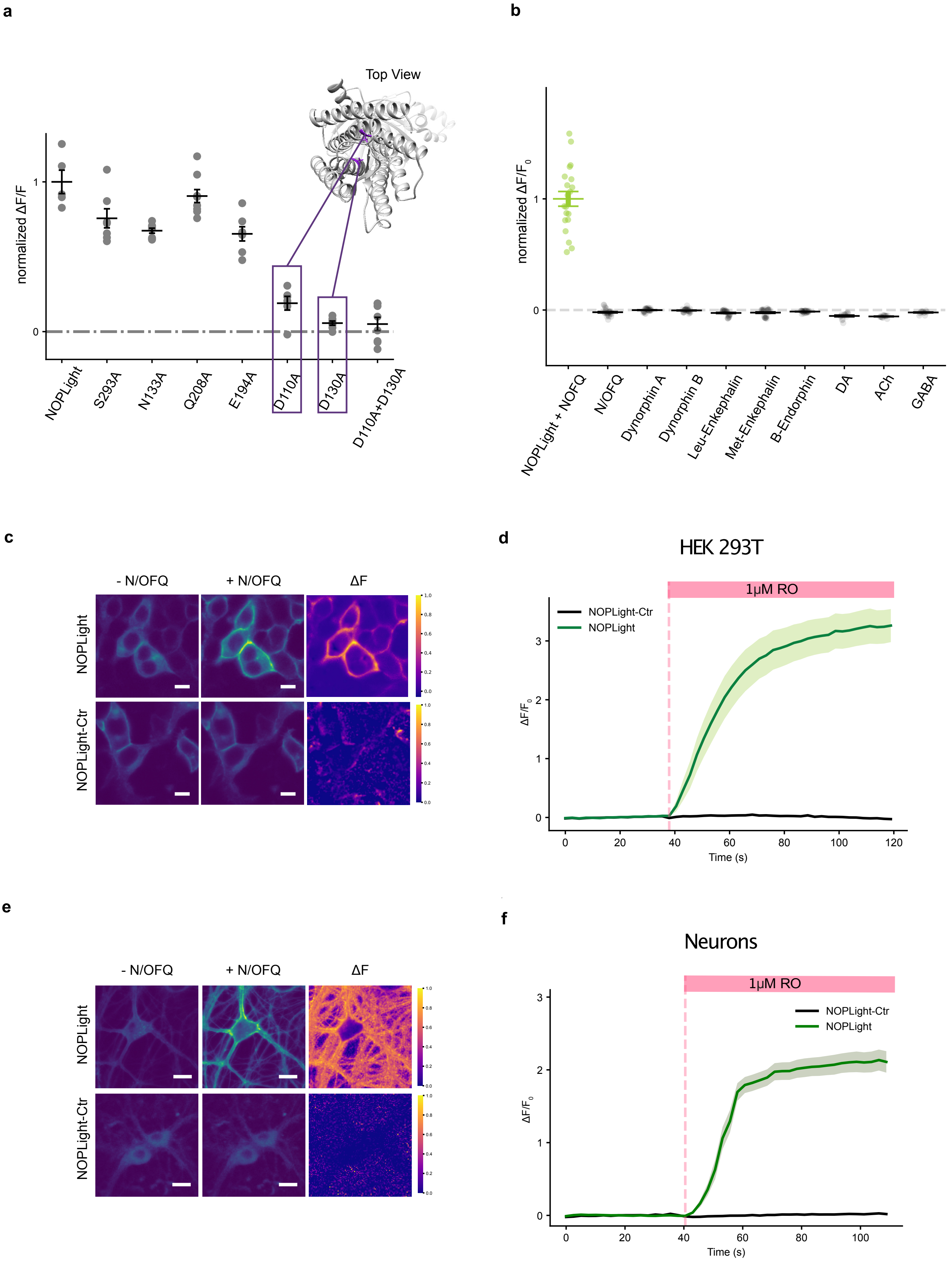

a
b
Top View
normalized ΔF/F
NOPLight
S293A
N133A
Q208A
E194A
D110A
D130A
D110A+D130A
normalized ΔF/F0
NOPLight + NOFQ
N/OFQ
Dynorphin A
Dynorphin B
Leu-Enkephalin
Met-Enkephalin
B-Endorphin
DA
ACh
GABA
c
- N/OFQ
+ N/OFQ
ΔF
NOPLight
NOPLight-Ctr
d
HEK 293T
1μM RO
NOPLight-Ctr
NOPLight
ΔF/F0
Time (s)
e
- N/OFQ
+ N/OFQ
ΔF
NOPLight
NOPLight-Ctr
f
Neurons
1μM RO
NOPLight-Ctr
NOPLight
ΔF/F0
Time (s)

Supplementary Figure 2. Development and characterisation of NOPLight-ctr.
a. Quantification of maximal $\Delta F/F_0$ in response to 10 μM N/OFQ in HEK 293T cells expressing NOPLight mutants. Mutated residues indicated by their absolute residue numbering in reference to human NOPR. Structural model of NOPLight-ctr is predicted by RoseTTAfold[78]. The two mutations selected for NOPLight-ctr are highlighted in purple. **b.** Normalized maximal $\Delta F/F_0$ of NOPLight-Ctr-expressing HEK 293T cells in response to endogenous opioid peptides (1 μM) and fast neurotransmitters (DA: dopamine, Ach: acetylcholine, GABA: gamma-Aminobutyric acid (1 mM)). All data normalized to NOPLight-expressing HEK293T cells (green). **c.** Representative images of HEK 293T cells expressing NOPLight (top; scale bars, 10 μm) and NOPLight-ctr (bottom; scale bars, 10 μm) before and after application of 1 μM Ro 64-6198. Corresponding normalized pixelwise ΔF shown on the right. **d.** Average fluorescent-fold change ($\Delta F/F_0$) of HEK 293T cell expressing NOPLight (light green trace) or NOPLight-ctr (black trace) in response to 1 μM RO 64-6198 (3 experiments, data shown as mean + SEM). **e.** Representative images of neurons expressing NOPLight (top; scale bars, 20 μm) and NOPLight-ctr (bottom; scale bars, 20 μm) before and after application of 1 μM Ro 64-6198. Corresponding normalized pixelwise ΔF shown on the right. **f.** Average fluorescent-fold change ($\Delta F/F_0$) of neurons expressing NOPLight (light green trace) or NOPLight-ctr (black trace) in response to 1 μM RO 64-6198 (3 experiments, data shown as mean ± SEM).

a **NOPLight Variants One-photon Spectra**

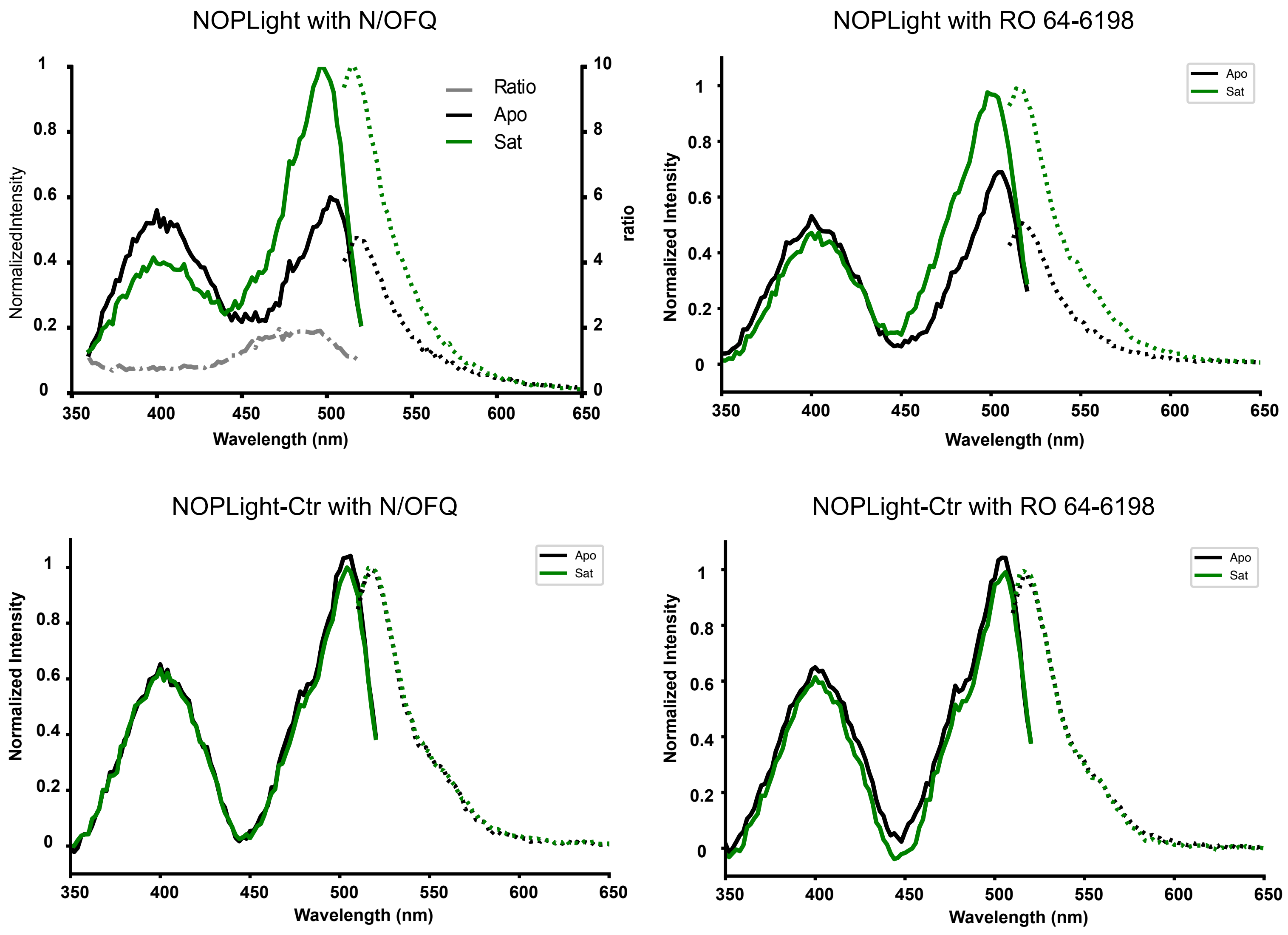

b **NOPLight Two-photon Spectra**

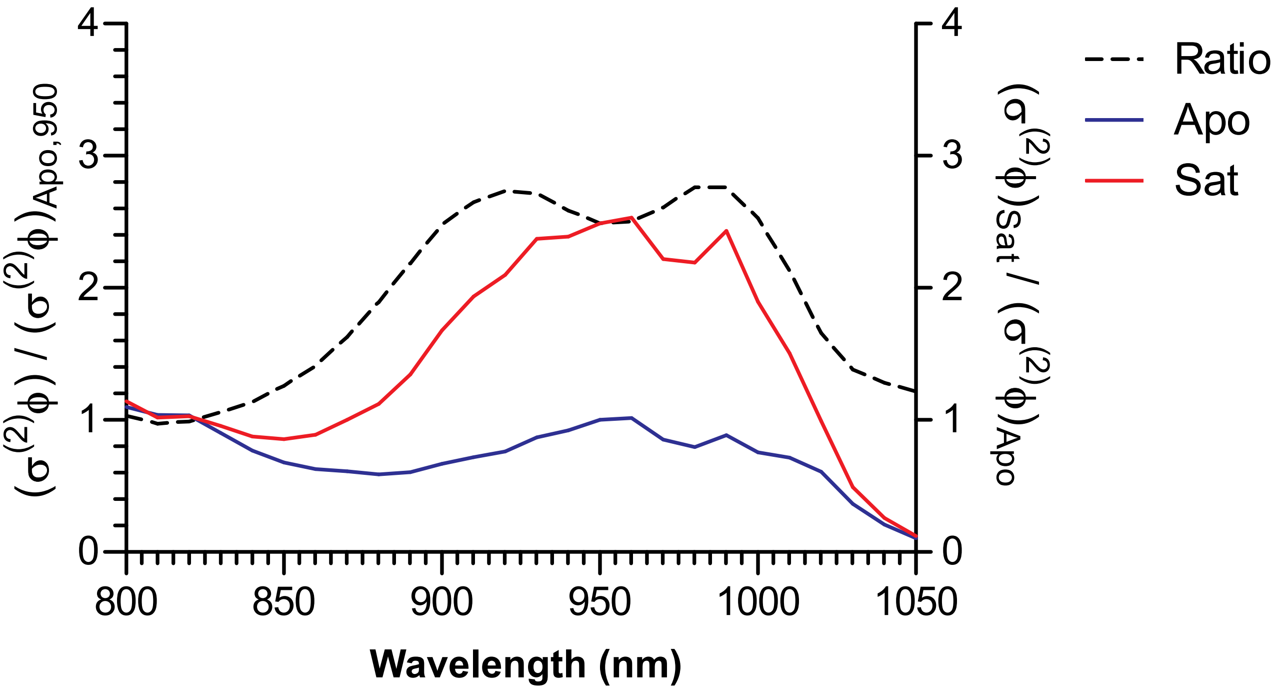

Supplementary Figure 3. Spectral properties of NOPLight and NOPLight-Ctr.
a. Top, normalized one-photon fluorescence excitation (solid lines, $\lambda_{excitation}$ = 360 – 520 nm, $\lambda_{emission}$ = 560 nm) and emission (dotted lines, $\lambda_{excitation}$ = 470 nm, $\lambda_{emission}$ = 510 – 650 nm) spectra of NOPLight-expressing HEK293T cells in the absence and presence of either N/OFQ (1 µM) or RO 64-6198 (1 µM). Traces shown are averaged from 3 independent experiments. Intensity measured at each wavelength is normalized to the maximum intensities measured for both the excitation and emission spectra. Bottom, same as on top with NOPLight-Ctr-expressing HEK 293T cells in the absence and presence of N/OFQ (1 µM) or RO 64-6198 (1 µM). **b.** Relative two-photon brightness of NOPLight imaged in transfected HEK cells grown attached to a glass coverslip in the presence (Sat) or absence (Apo) of N/OFQ (1 µM). Ratio between Sat and Apo shown in dashed line. Each trace is the average of 3 independent experiments.

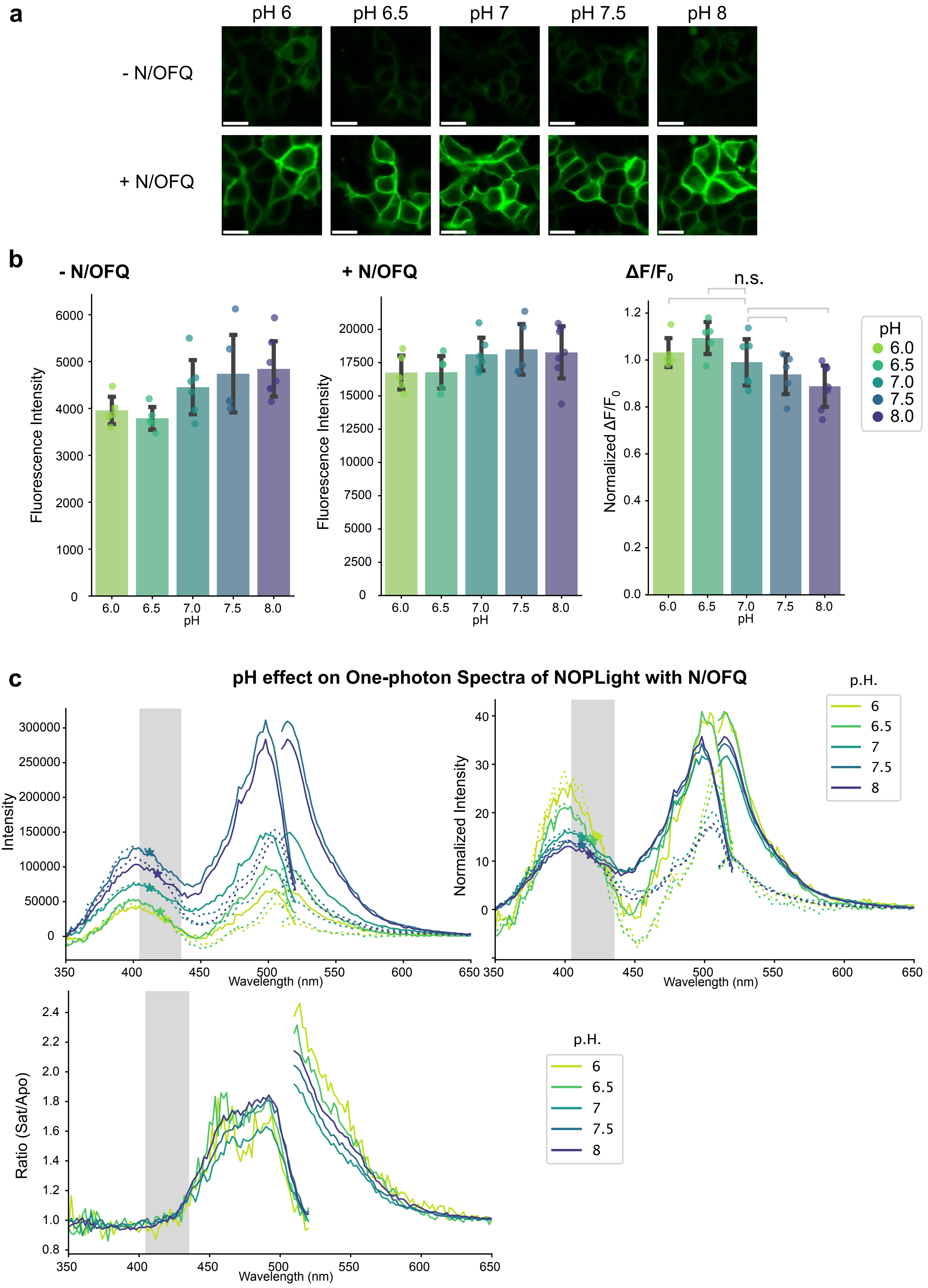

a
pH 6
pH 6.5
pH 7
pH 7.5
pH 8
- N/OFQ
+ N/OFQ
b
- N/OFQ
+ N/OFQ
ΔF/F0
n.s.
Fluorescence Intensity
Normalized ΔF/F0
pH
6.0
6.5
7.0
7.5
8.0
c
pH effect on One-photon Spectra of NOPLight with N/OFQ
Intensity
Normalized Intensity
Ratio (Sat/Apo)
Wavelength (nm)
p.H.
6
6.5
7
7.5
8

Supplementary Figure 4. Characterization of NOPlight sensitivity to external p.H.
a. Representative images of NOPLight-expressing HEK 293T cells in the absence (top) and presence (bottom) of 1 μM N/OFQ. Scale bar: 20 μm. **b.** Quantification of fluorescence intensity in the absence (left) and presence (middle) of 1 μM N/OFQ, and fluorescent-fold change (ΔF/F) normalized to the ΔF/F measured at extracellular p.H. 7 (right, ANOVA with Tukey Kramer post-hoc test of all p.H. conditions compared to p.H, 7. P = 0. 942, 0.358, 0.883 and 0.289 respectively). Data shown as mean ± SEM. **c.** Top left: One-photon spectra of NOPLight-expressing HEK 293T cells at various extracellular p.H. in the presence (Sat, solid lines) and absence (Apo, dashed lines) of N/OFQ (1 μM). Isosbestic point at each p.H. marked with “ * ”. Top right: One-photon spectra of NOPLight-expressing HEK 293T cells at various p.H. normalized by the corresponding area under the curve of with the presence of N/OFQ at each extracellular p.H. condition. Bottom: Ratio between Sat and Apo at various extracellular p.H.. Shaded bar indicates the range between 405 to 435 nm. Each trace is the average of 3 independent experiments.

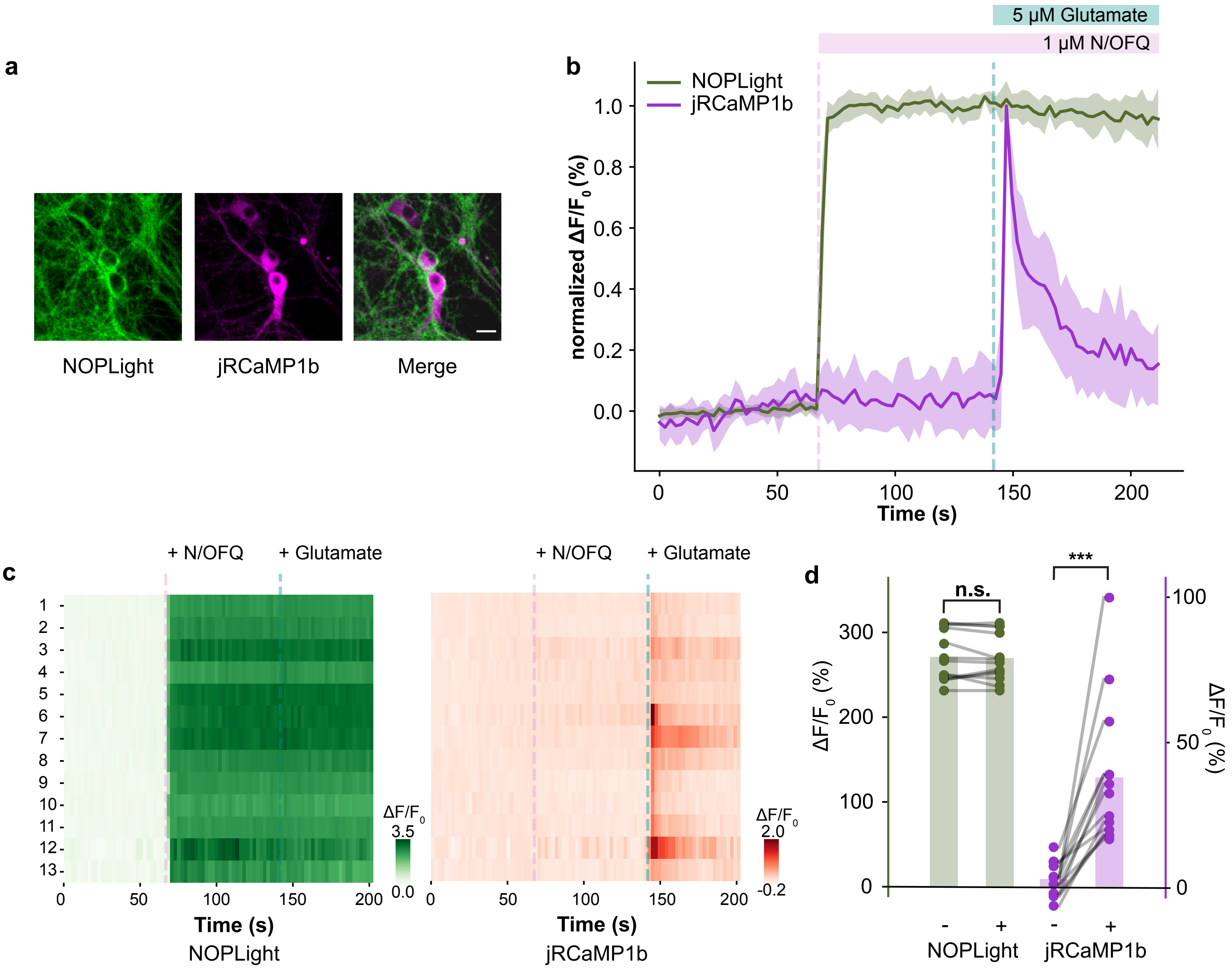
a
NOPLight
jRCaMP1b
Merge
b
5 μM Glutamate
1 μM N/OFQ
NOPLight
jRCaMP1b
normalized ΔF/F0 (%)
1.0
0.8
0.6
0.4
0.2
0.0
0
50
100
150
200
Time (s)
c
+ N/OFQ
+ Glutamate
1
2
3
4
5
6
7
8
9
10
11
12
13
ΔF/F0
3.5
0.0
2.0
-0.2
Time (s)
NOPLight
jRCaMP1b
d
n.s.
***
ΔF/F0 (%)
300
200
100
0
100
50
-
+
NOPLight
jRCaMP1b

Supplementary Figure 5. Changes in neuronal activity do not affect the fluorescence response of NOPLight to N/OFQ.

a. Representative image of neurons co-expressing NOPLight (right) and jRCaMP1b (middle). Scale bar: 20 μm. **b.** Normalized average fluorescent-fold change ($\Delta F/F_0$) of NOPLight (green) and jRCaMP1b (magenta) in response to 1 μM N/OFQ followed by 5 μM glutamate. (13 neurons from 2 independent experiments, data shown as mean ± SD). **c.** Corresponding heat map of **b**, where each row corresponds to one neuron. Left: NOPLight fluorescent response; Right: jRCaMP1b fluorescent response. **d.** Quantification of average $\Delta F/F_0$ (%) from **b**. NOPLight (left Y-axis, green) and jRCaMP1b (right Y-axis, magenta) signals were quantified 30 seconds before (-) and after (+) application of glutamate (paired sample t-test, ***P<0.001, P=0.4370, NOPLight, P=0.0002, jRCaMP1b).

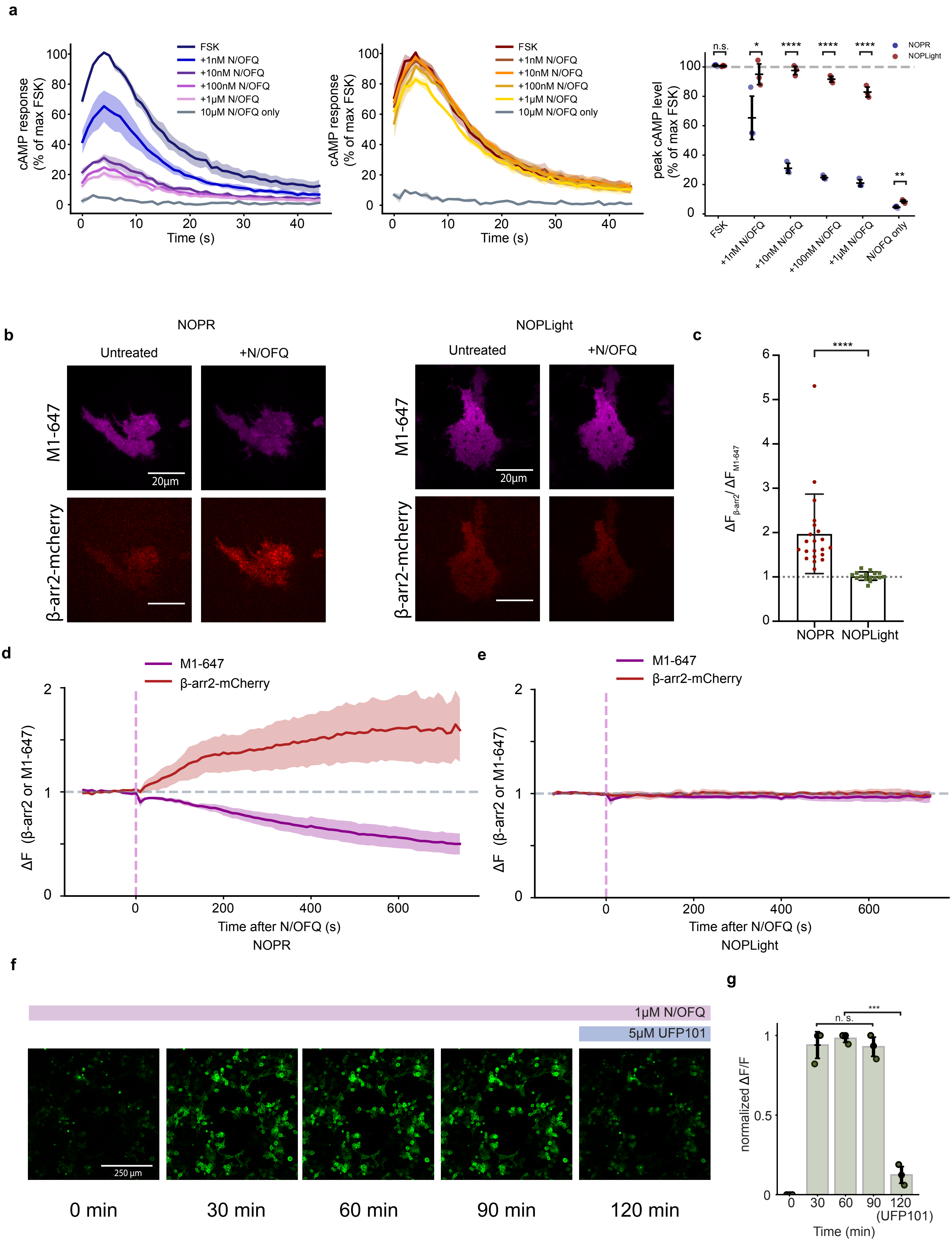

a
cAMP response (% of max FSK)
Time (s)
FSK
+1nM N/OFQ
+10nM N/OFQ
+100nM N/OFQ
+1μM N/OFQ
10μM N/OFQ only
peak cAMP level (% of max FSK)
NOPR
NOPLight
n.s.
*
****
**
FSK
+1nM N/OFQ
+10nM N/OFQ
+100nM N/OFQ
+1μM N/OFQ
N/OFQ only
b
NOPR
NOPLight
Untreated
+N/OFQ
M1-647
β-arr2-mcherry
20μm
c
NOPR
NOPLight
d
M1-647
β-arr2-mCherry
ΔF (β-arr2 or M1-647)
Time after N/OFQ (s)
NOPR
e
NOPLight
f
1μM N/OFQ
5μM UFP101
250 μm
0 min
30 min
60 min
90 min
120 min
g
n. s.
***
normalized ΔF/F
(UFP101)
Time (min)

Supplementary Figure 6. Characterization of sensor coupling to intracellular signaling pathways.

a. Left: cAMP GloSensor luciferase activity traces in NOPR expressing HEK293 cells in response to different concentrations of N/OFQ, normalized to the peak activity evoked by 3 μM of Forskolin (FSK). Middle: cAMP GloSensor luciferase activity traces in NOPLight expressing HEK293 cells in response of different concentrations of N/OFQ, normalized to the peak activity evoked by 3 μM of Forskolin (FSK). Right: Quantification of peak cAMP activity of NOPR and NOPLight expressing cells at different concentrations of N/OFQ. (n = 3 independent experiments, data shown as mean ± SEM, two sample t-test, n.s. P = 0.584, *P = 0.0623, **P = 0.00874, ****P < 0.0001). **b**. Representative images of cells co-expressing mCherry-tagged β-arrestin-2 with NOPR (left, scale bar = 20 μm) or NOPLight (right, scale bar = 20 μm) before and 12.5 min after N/OFQ (10 μM) stimulation. NOPR and NOPLight are labeled with Alexa-647-conjugated M1 anti-FLAG antibody (M1-647). **c**. Ratio of change in mCherry-tagged β-arrestin-2 signal versus change of M1-647 signal in NOPR and NOPLight expressing cells. (n = 21 and 16 cells respectively, from 3 independent experiments, data shown as mean ± SD, one-sided Mann-Whitney U test, P<0.0001). **d**. Plasma membrane signal (TIRF) of mCherry-tagged β-arrestin-2 and FLAG-tagged NOPR signal before and after the application of N/OFQ. (n = 3 independent experiments, data shown as mean ± s.e.m) **e.** Similar to **d**, cells expressing NOPLight instead of NOPR. **f.** representative images of NOPLight expressing HEK293T cells at 0 min, 30 min, 60 min, 90 min and 120 min after the addition of N/OFQ (1μM). 5 μM UFP 101 was added at t = 110 min. **g.** Normalized fluorescent response ($\Delta F/F_0$) at time points as in **f.** (n = 3 independent experiments, P = 0.7522 for one-way repeated measures ANOVA test between fluorescence response at 30, 60 and 120 min after N/OFQ, P<0.001 for one-way repeated measures ANOVA test between 30, 60, 120 min after N/OFQ and after UFP 101.).

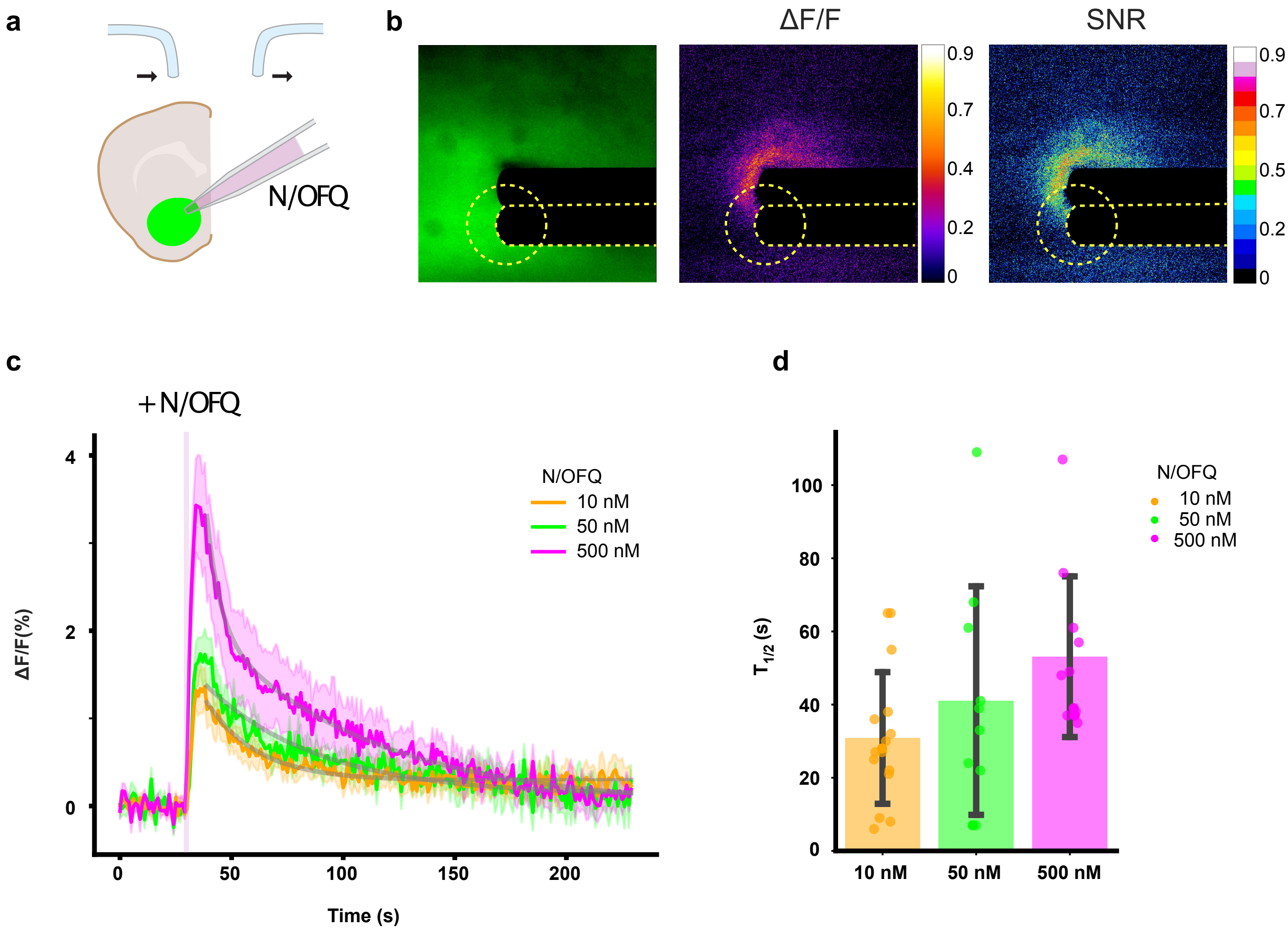

a
N/OFQ
b
ΔF/F
SNR
0.9
0.7
0.4
0.2
0
0.9
0.7
0.5
0.2
0
c
+N/OFQ
ΔF/F(%)
Time (s)
N/OFQ
10 nM
50 nM
500 nM
d
T1/2 (s)
N/OFQ
10 nM
50 nM
500 nM

Supplementary Figure 7. NOPlight kinetic measurements in brain slices.
a. Schematic representation of AAV-DJ-hSynapsin1-NOPLight injection into the NAc. **b.** Left: Representative image of acute brain slice expressing NOPLight; middle: pixel-wise ΔF/F immediately after application of N/OFQ; right: pixel-wise signal to noise ratio immediately after application of N/OFQ. **c.** Time-course plot of ΔF/F traces depicting the response under each concentration of N/OFQ. Data shown as mean (solid line) ± SEM (shaded area). Gray traces represent fitted mono-exponential decay model. Shaded pink bar represents the time point at which N/OFQ was applied. n = 16, 10, 11 slices respectively with 10, 50 and 500 nM N/OFQ from N = 5 animals. **d.** Quantification of the decay half-time at different concentrations of N/OFQ. n = 16, 10, 11 slices respectively. Bar plot represents mean ± SEM.

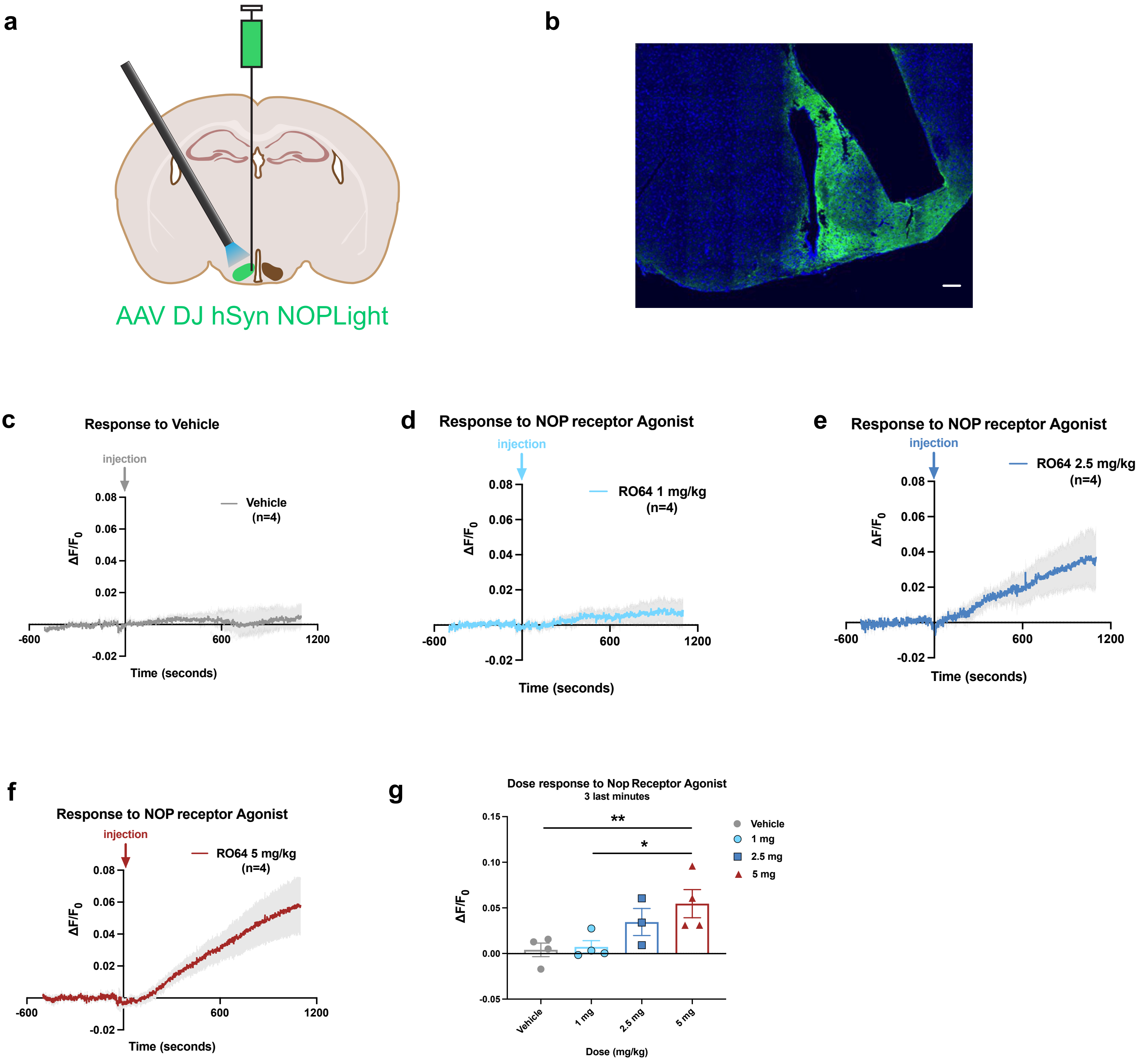

a
AAV DJ hSyn NOPLight
b
c
Response to Vehicle
injection
Vehicle
(n=4)
ΔF/F0
Time (seconds)
d
Response to NOP receptor Agonist
injection
RO64 1 mg/kg
(n=4)
e
Response to NOP receptor Agonist
injection
RO64 2.5 mg/kg
(n=4)
f
Response to NOP receptor Agonist
injection
RO64 5 mg/kg
(n=4)
g
Dose response to Nop Receptor Agonist
3 last minutes
Vehicle
1 mg
2.5 mg
5 mg
**
*
Dose (mg/kg)

Supplementary Figure 8. Target engagement of a NOPR agonist in the arcuate nucleus. **a.** Schematic representation showing AAV-DJ-hSynapsin1-NOPLight (NOPsyn) (0.8 x 1013 GC/mL) injection into the arcuate nucleus of the hypothalamus (ARC), followed by optic fibre implantation. **b.** Representative image of NOPLight-expressing brain slice showing optic fibre placement. GFP (green) expression, and DAPI in the ARC. Scale bar, 100 µm. **c**. Average *in vivo* photometry trace ($\Delta F/F_0$) for vehicle response. **d**. Average fluorescence traces showing the response to RO64 injection at 1 mg/kg. **e**. Average fluorescence traces showing the response to RO64 at 2.5 mg/kg. **f**. Average fluorescence traces showing the response to RO64 at 5 mg/kg. All injections were performed I.P. **g**. Quantification of average fluorescence signals in 3-min bins (957-1137 s) at the end of fibre photometry recordings, including data from (**c-f**) vehicle response and the response to RO64 (1, 2.5, and 5 mg/kg) IP injection. n=5 mice. Data are expressed as mean ± SEM. One-way ANOVA with Tukey's multiple comparisons test. Vehicle vs. RO64 5 mg/kg **P= 0.0058. RO64 1 mg/kg vs. RO64 5 mg/kg *P= 0.0131.

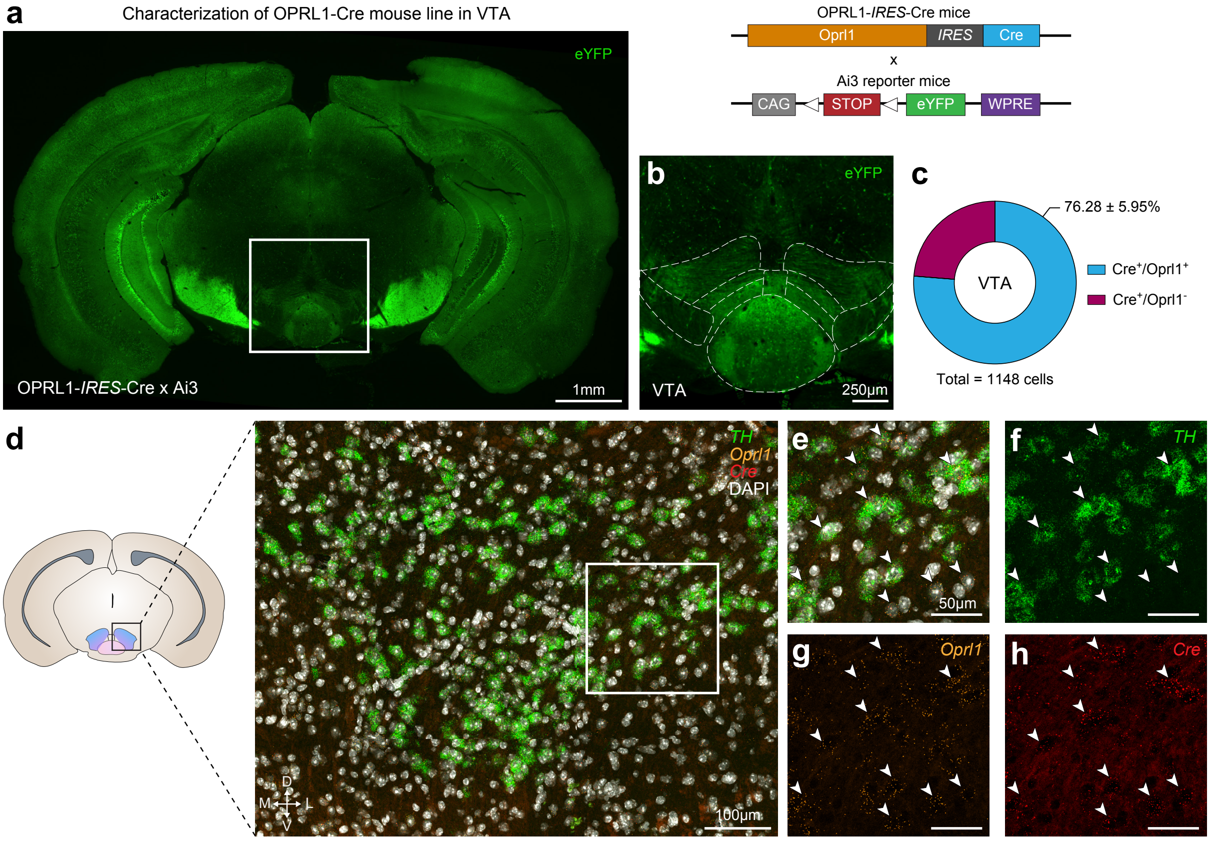

a
Characterization of OPRL1-Cre mouse line in VTA
eYFP
OPRL1-IRES-Cre x Ai3
1mm
OPRL1-IRES-Cre mice
Oprl1
IRES
Cre
x
Ai3 reporter mice
CAG
STOP
eYFP
WPRE
b
eYFP
VTA
250µm
c
76.28 ± 5.95%
VTA
Cre+/Oprl1+
Cre+/Oprl1-
Total = 1148 cells
d
TH
Oprl1
Cre
DAPI
D
M
L
V
100µm
e
50µm
f
TH
g
Oprl1
h
Cre

Supplementary Figure 9. Anatomical characterization of the OPRL1-Cre mouse line in the ventral tegmental area.

a. An OPRL1-Cre mouse line was generated and crossed with an Ai3 reporter line. Coronal image at bregma -3.28 mm showing Ai3-eYFP expression driven by OPRL1-Cre. **b.** Higher magnification of inset from **a** showing eYFP reporter expression in the ventral tegmental area (VTA). **c.** Quantification of *Oprl1* expression within Cre-expressing cells detected by *in situ* hybridization in the VTA (n = 2; 2 mice, 2 and 3 slices/mouse). Data represented as mean ± SEM. **d.** Representative image showing *Oprl1* and Cre expression patterns in Oprl1-Cre mice via *in situ* hybridization of *Oprl1* (orange), Cre (red), *Th* (tyrosine hydroxylase, green), and DAPI (white) in the VTA. **e-h.** Higher magnification of inset from **d** showing combined (**e**) and individual (**f-h**) channels for *Th*, *Oprl1*, Cre, and DAPI. Scale bars, 50 µm.

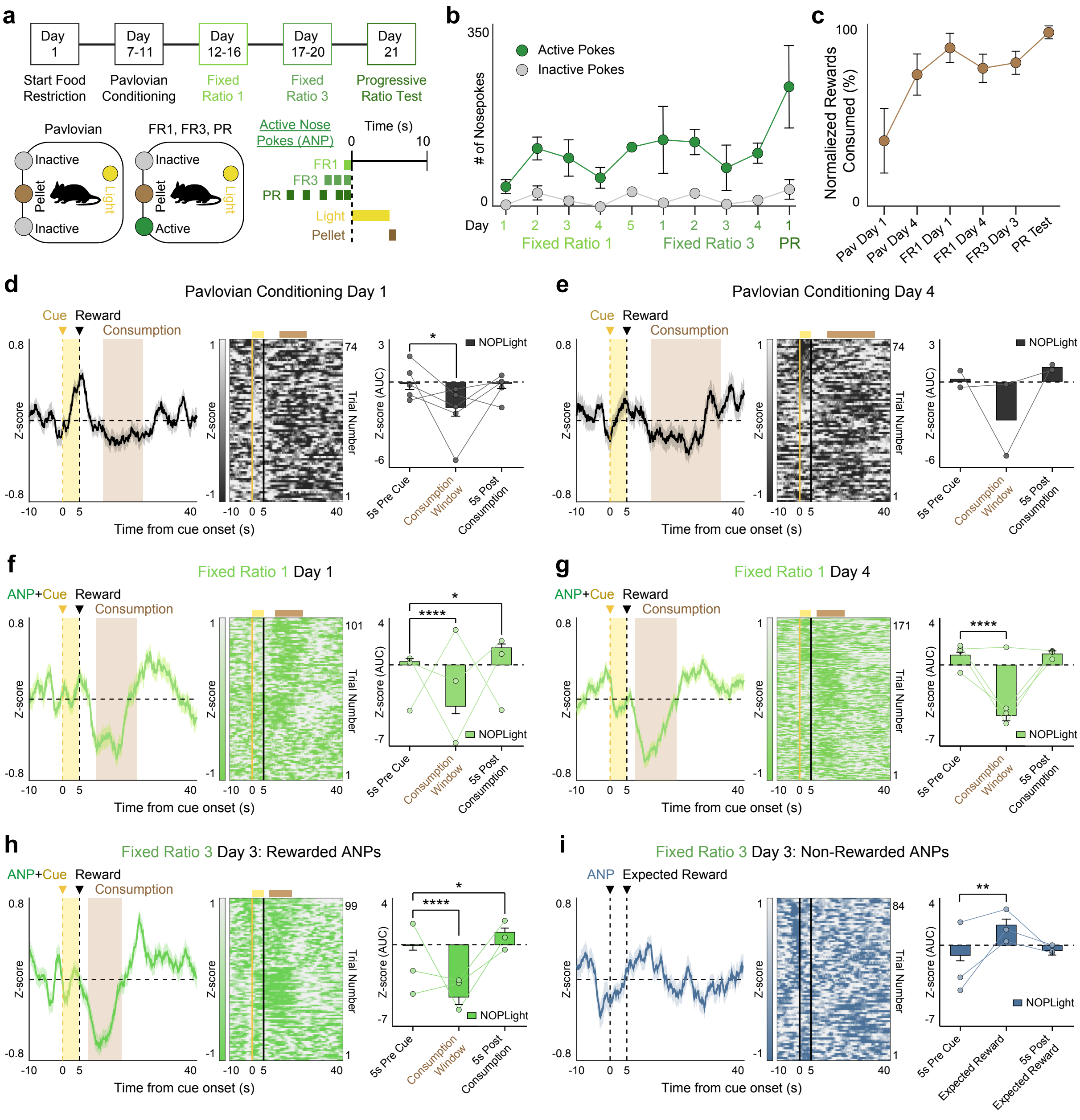
a
Day 1
Day 7-11
Day 12-16
Day 17-20
Day 21
Start Food Restriction
Pavlovian Conditioning
Fixed Ratio 1
Fixed Ratio 3
Progressive Ratio Test
Pavlovian
FR1, FR3, PR
Inactive
Pellet
Light
Active
Active Nose Pokes (ANP)
Time (s)
FR1
FR3
PR
Pellet
b
# of Nosepokes
Active Pokes
Inactive Pokes
Day
Fixed Ratio 1
Fixed Ratio 3
PR
c
Normalieszed Rewards Consumed (%)
Pav Day 1
Pav Day 4
FR1 Day 1
FR1 Day 4
FR3 Day 3
PR Test
d
Pavlovian Conditioning Day 1
Cue
Reward
Consumption
Z-score
Trial Number
Z-score (AUC)
NOPLight
5s Pre Cue
Consumption Window
5s Post Consumption
Time from cue onset (s)
e
Pavlovian Conditioning Day 4
f
Fixed Ratio 1 Day 1
ANP+Cue
g
Fixed Ratio 1 Day 4
h
Fixed Ratio 3 Day 3: Rewarded ANPs
i
Fixed Ratio 3 Day 3: Non-Rewarded ANPs
ANP
Expected Reward
5s Pre Cue
Expected Reward
5s Post Expected Reward

Supplementary Figure 10. NOPLight detection of endogenous VTA N/OFQ release during Pavlovian and operant conditioning.

a. Mice expressing NOPLight in the VTA were trained on Pavlovian and fixed ratio schedules prior to progressive ratio test (n = 5 mice). Top: Training regimen for conditioning. Bottom: Cartoon depicting operant box setup and trial structure. **b.** Total number of nose-pokes made in active (green) or inactive (grey) nose-poke ports across training days. Data represented as mean ± SEM. **c.** Proportion of delivered rewards that animals consumed on photometry recording days. Data represented as mean ± SEM. **d-e.** Left: Trace of mean NOPLight signal in the first (**d**) and fourth (**e**) Pavlovian conditioning sessions, aligned to light cue onset (yellow, shaded). Time to pellet retrieval and duration of consumption averaged across trials (brown, shaded). Middle: Corresponding heat map. Right: Area under the curve (AUC) for photometry traces from **d** and **e** respectively, calculated over the averaged reward consumption window and 5-second intervals surrounding the consumption period (**d:** two-tailed Wilcoxon test, *p=0.0364, n = 5 mice, data represented as mean ± SEM; **e:** n = 2 mice). **f-g.** Same as (**d-e**) but for the first (**f**) and fourth (**g**) fixed ratio 1 (FR1) sessions (two-tailed Wilcoxon test, *p=0.0105, ****p<0.0001, n = 3 mice). Data represented as mean ± SEM. **h.** Left: Trace of mean NOPLight signal during fixed ratio 3 (FR3) training, aligned to reinforced active nose-pokes. Shown with averaged time to pellet retrieval and duration of consumption (brown, shaded). Middle: Corresponding heat map. Right: AUC of photometry trace calculated over the averaged consumption window and in 5-second intervals surrounding the consumption period (two-tailed Wilcoxon test, *p=0.0477, ****p<0.0001, n = 3 mice). Data represented as mean ± SEM. **i.** Left: Trace of mean NOPLight signal during the same FR3 session as **h**, aligned to non-rewarded active nose-pokes. Middle: Corresponding heat map. Right: AUC of photometry trace calculated over the expected reward period and in 5-second intervals surrounding expected reward (two-tailed Wilcoxon test, **p=0.0017, n = 3 mice). Data represented as mean ± SEM.
