# Supplementary material for: Development of a genetically encoded sensor for probing endogenous nociceptin opioid peptide release
Source: Nat Commun. 2024 Jun 25;15:5353. doi: 10.1038/s41467-024-49712-0 (PMC11199706; doi:10.1038/s41467-024-49712-0)
Supplement: Supplementary file 3 — Reporting Summary [file 41467_2024_49712_MOESM3_ESM.pdf]

nature portfolio

Corresponding author(s): Tommaso Patriarchi, Michael R. Bruchas

Last updated by author(s): May 27, 2024

# Reporting Summary

Nature Portfolio wishes to improve the reproducibility of the work that we publish. This form provides structure for consistency and transparency in reporting. For further information on Nature Portfolio policies, see our Editorial Policies and the Editorial Policy Checklist.

## Statistics

For all statistical analyses, confirm that the following items are present in the figure legend, table legend, main text, or Methods section.

| n/a | Confirmed | |
|---|---|---|
| ☐ | ☒ | The exact sample size (*n*) for each experimental group/condition, given as a discrete number and unit of measurement |
| ☐ | ☒ | A statement on whether measurements were taken from distinct samples or whether the same sample was measured repeatedly |
| ☐ | ☒ | The statistical test(s) used AND whether they are one- or two-sided<br>*Only common tests should be described solely by name; describe more complex techniques in the Methods section.* |
| ☒ | ☐ | A description of all covariates tested |
| ☒ | ☐ | A description of any assumptions or corrections, such as tests of normality and adjustment for multiple comparisons |
| ☐ | ☒ | A full description of the statistical parameters including central tendency (e.g. means) or other basic estimates (e.g. regression coefficient) AND variation (e.g. standard deviation) or associated estimates of uncertainty (e.g. confidence intervals) |
| ☐ | ☒ | For null hypothesis testing, the test statistic (e.g. *F*, *t*, *r*) with confidence intervals, effect sizes, degrees of freedom and *P* value noted<br>*Give P values as exact values whenever suitable.* |
| ☒ | ☐ | For Bayesian analysis, information on the choice of priors and Markov chain Monte Carlo settings |
| ☒ | ☐ | For hierarchical and complex designs, identification of the appropriate level for tests and full reporting of outcomes |
| ☒ | ☐ | Estimates of effect sizes (e.g. Cohen's *d*, Pearson's *r*), indicating how they were calculated |

*Our web collection on statistics for biologists contains articles on many of the points above.*

## Software and code

Policy information about availability of computer code

| | |
|---|---|
| Data collection | Below is the software used for data collection in this manuscript:<br>• Zeiss Zen Blue (https:/ /www.zeiss.com/microscopy/int/products/microscope-software/zen-lite.html)<br>• MATLAB versions R2019b (https://www.mathworks.com/products/matlab.html) |
| Data analysis | Below is the software used for data analysis in this manuscript:<br>• ImageJ version 1.52 (http://imagej.nih.gov/ij/download.html)<br>• GraphPad Prism 9.0.0 (https://www.graphpad.com/scientific-software/prism/)<br>• MATLAB versions R2019b (https://www.mathworks.com/products/matlab.html) |

For manuscripts utilizing custom algorithms or software that are central to the research but not yet described in published literature, software must be made available to editors and reviewers. We strongly encourage code deposition in a community repository (e.g. GitHub). See the Nature Portfolio guidelines for submitting code & software for further information.

## Data

Policy information about availability of data

All manuscripts must include a data availability statement. This statement should provide the following information, where applicable:

- Accession codes, unique identifiers, or web links for publicly available datasets
- A description of any restrictions on data availability
- For clinical datasets or third party data, please ensure that the statement adheres to our policy

DNA and protein sequences for the sensors developed in this study have been deposited on the National Center for Biotechnology Information database (accession nos. OQ067483 https://www.ncbi.nlm.nih.gov/nuccore/OQ067483.1/ and OQ067484 https://www.ncbi.nlm.nih.gov/nuccore/OQ067484.1/ ) and are available in Supplementary Note S1. DNA plasmids used for viral production have been deposited both on the UZH Viral Vector Facility (https://vvf.ethz.ch/) and on Addgene (NOPLight: Addgene plasmid # 195578, http://n2t.net/addgene:195578; NOPLight-Ctr: Addgene plasmid # 195579, http://n2t.net/addgene:195579). Viral vectors can be obtained either from the Patriarchi laboratory, the UZH Viral Vector Facility, or Addgene. All source data are provided with the manuscript. Raw data is available at https://zenodo.org/doi/10.5281/zenodo.11149714 or by emailing the corresponding authors.

## Research involving human participants, their data, or biological material

Policy information about studies with human participants or human data. See also policy information about sex, gender (identity/presentation), and sexual orientation and race, ethnicity and racism.

| | |
|---|---|
| Reporting on sex and gender | N/A |
| Reporting on race, ethnicity, or other socially relevant groupings | N/A |
| Population characteristics | N/A |
| Recruitment | N/A |
| Ethics oversight | N/A |

Note that full information on the approval of the study protocol must also be provided in the manuscript.

# Field-specific reporting

Please select the one below that is the best fit for your research. If you are not sure, read the appropriate sections before making your selection.

☒ Life sciences ☐ Behavioural & social sciences ☐ Ecological, evolutionary & environmental sciences

For a reference copy of the document with all sections, see nature.com/documents/nr-reporting-summary-flat.pdf

# Life sciences study design

All studies must disclose on these points even when the disclosure is negative.

| | |
|---|---|
| Sample size | No sample size calculation was performed. Sample sizes were based on the previous scientific literature in the field (Duffet et al, Nat Methods 2022; Patriarchi et al, Nat Methods 2020; Patriarchi et al, Science 2018). |
| Data exclusions | In the VTA exogenous NOPR agonism dose response experiment (Figure 3c), 3 animals were excluded for off-target fiber placement after post-hoc validation via immunohistochemistry, as in previous literature (Siuda et al, Neuron 2015; Al-Hasani et al, Nat Neurosci 2021). |
| Replication | All experiments were repeated at least three independent times using multiple cell cultures and multiple animal subjects within each group. All replication attempts were successful. |
| Randomization | Group allocations used in this study were randomly assigned to animals and/or cultured cells. |
| Blinding | Blinding during group allocations was not adopted in this study and data analysis was performed in an objective and unbiased fashion, as in previous literature (Duffet et al, Nat Methods 2022; Patriarchi et al, Nat Methods 2020). |

# Reporting for specific materials, systems and methods

We require information from authors about some types of materials, experimental systems and methods used in many studies. Here, indicate whether each material, system or method listed is relevant to your study. If you are not sure if a list item applies to your research, read the appropriate section before selecting a response.

## Materials & experimental systems

| n/a | Involved in the study |
|---|---|
| ☐ | ☒ Antibodies |
| ☐ | ☒ Eukaryotic cell lines |
| ☒ | ☐ Palaeontology and archaeology |
| ☐ | ☒ Animals and other organisms |
| ☒ | ☐ Clinical data |
| ☒ | ☐ Dual use research of concern |
| ☒ | ☐ Plants |

## Methods

| n/a | Involved in the study |
|---|---|
| ☒ | ☐ ChIP-seq |
| ☒ | ☐ Flow cytometry |
| ☒ | ☐ MRI-based neuroimaging |

# Antibodies

Antibodies used

chicken anti-GFP, 1:1000, ab13970, Abcam; rat anti-mcherry, 1:1000, Thermo Fisher Scientific, M11217; goat anti-chicken-FITC, Jackson #103-095-155, 1:500; goat anti-rabbit Alexa-Fluor-594, Thermo Fisher Scientific, A11012; 1:500; chicken anti-GFP, 1:2000, Abcam #ab92456; AlexaFluor 488 goat anti-chicken, Abcam #ab150169.

Validation

All antibodies were validated by the commercial provider.

# Eukaryotic cell lines

Policy information about cell lines and Sex and Gender in Research

Cell line source(s)

HEK293T cells (ATCC cat#3216)

Authentication

The cell lines were authenticated by the vendor (ATCC) using Short Tandem Repeat (STR) Profiling to detect misidentified, cross-contaminated, or genetically-drifted lines.

Mycoplasma contamination

The cell line used was mycoplasma-free.

Commonly misidentified lines (See ICLAC register)

The study did not involve commonly misidentified cell lines.

# Animals and other research organisms

Policy information about studies involving animals; ARRIVE guidelines recommended for reporting animal research, and Sex and Gender in Research

Laboratory animals

Rat embryos (E17) obtained from timed-pregnant Wistar rats (Envigo) were used for preparing primary cortical neuronal cultures. 10-24 week-old mice of the following strains were used in this study: PNOC-Cre mice (Jackson Research Labs: strain #:034278); PNOC-Cre mice: PMID: 32302532; OPRL1-Cre mice (Jackson Research Labs: strain #:036308); wild-type C57/Bl6.

Wild animals

N/A

Reporting on sex

Post-hoc sex-based analysis was not conducted due to sample size. The purpose of this study was to develop a tool and demonstrate its functionality in sensing endogenous opioid peptide dynamics in vivo using a design strategy and viral vector well-established for use in both sexes. The number of genetic lines/mice, sensor variants, testing parameters, and appropriate controls necessary in the experiments along with the frequency of trouble shooting involved in a tool development project made sufficiently powering for sex-based analysis both resource and cost prohibitive. Males and females were used in pharmacology and chemogenetic studies, and sensor expression and functionality were detectable in all animals. Circuit activity during the reward-related behaviors used in the study has been extensively characterized previously to be non sex-dependent (Parker and Pedersen et al., Cell 2019). For stress behavior, our preliminary findings with GCaMP showed differences in the stress sensitivity of these neurons in males and females, with males having higher sensitivity. Signal to noise detection is a critical aspect of tool development and characterization for new sensors, so males were used in the tail suspension studies. Sexes used in each experiment are also reported in the methods.

Field-collected samples

N/A

Ethics oversight

Animal procedures were approved and performed following the local government authorities (Bezirksregierung Köln, Animal Care and Use Committee of University of Washington).

Note that full information on the approval of the study protocol must also be provided in the manuscript.
